# Antenatal Diagnosis of a True Knot of the Umbilical Cord: A Rare Case With a Successful Surgical Outcome

**DOI:** 10.7759/cureus.86095

**Published:** 2025-06-15

**Authors:** Sarmistha Gupta, Rajalakshmi Srinivasan

**Affiliations:** 1 Radiology, Medcare Royal Speciality Hospital, Dubai, ARE; 2 Obstetrics and Gynecology, Medcare Royal Speciality Hospital, Dubai, ARE

**Keywords:** antenatal diagnosis, doppler ultrasound, emergency cesarean section, fetal distress, true umbilical cord knot

## Abstract

True knots of the umbilical cord (TKUCs) are rare and can compromise fetal well-being. Their diagnosis is typically made at delivery, with antenatal identification remaining rare. This case report describes an antenatal diagnosis of a TKUC at 35 weeks’ gestation in a 28-year-old third gravida with two previous miscarriages. The patient presented with reduced amniotic fluid and absent fetal movements. Ultrasound imaging raised suspicion of a TKUC, prompting an emergency cesarean section. A healthy neonate was delivered. This case highlights the potential role of color Doppler ultrasound and maternal vigilance in identifying TKUCs before delivery. Despite diagnostic challenges, heightened clinical awareness and timely intervention can contribute to favorable perinatal outcomes. Future research should aim to improve diagnostic accuracy and develop effective risk-stratification tools.

## Introduction

True knots of the umbilical cord (TKUCs) are a rare but clinically significant obstetric event, occurring in approximately 0.3% to 2.1% of pregnancies [1,2]. While many knots remain asymptomatic and go undetected, tight knots can compromise fetal circulation, leading to complications like hypoxia, intrauterine growth restriction, or stillbirth, with perinatal mortality increasing up to fourfold [2-4]. True knots are believed to form between 9 and 12 weeks of gestation [2,5], likely influenced by increased fetal mobility and abundant amniotic fluid during early pregnancy.

Antenatal identification of TKUC continues to pose a significant diagnostic challenge, despite advances in imaging technology, primarily due to the dynamic nature of the cord and the limited sensitivity of conventional ultrasound, with detection rate reported as low as 12% [2,3]. Nonetheless, early diagnosis is crucial, as it enables timely and potentially life-saving obstetric interventions aimed at reducing perinatal morbidity and mortality.

Advanced imaging modalities such as color Doppler, power Doppler, and 3D sonography have improved the antenatal detection of TKUCs [3,6]. However, false positives in these techniques may arise due to misinterpretation of cord configurations or other abnormalities that mimic malformations, most of which do not require intervention [1,6]. False knots, more accurately termed "nodus spurious vasculosis," represent benign vascular redundancies of umbilical vessels and do not pose clinical risk [6]. These can mimic true knots on ultrasound, but advanced modalities like color Doppler and 3D imaging allow for clear differentiation based on characteristic findings [6].

TKUCs are associated with several maternal and fetal risk factors, including advanced maternal age, multiparity, polyhydramnios, long umbilical cords, and small-for-gestational-age (SGA) fetuses [2]. Several studies have identified male fetal sex as the most significant risk factor [4,7,8], likely attributed to the generally longer umbilical cords observed in male fetuses [7,8].

This case report presents a rare incidence of antenatal diagnosis of a TKUC, confirmed intraoperatively. The case underscores current diagnostic tools' limitations, imaging's potential role, and the need for heightened clinical awareness in high-risk pregnancies. These gaps underscore the need for prospective studies evaluating the diagnostic accuracy of imaging techniques, development of standardized management protocols, and further exploration into the natural history and clinical impact of TKUC when diagnosed antenatally. By sharing this experience, we aim to contribute to the limited body of literature addressing antenatal identification of TKUC and highlight its implications for clinical management and fetal outcomes.

## Case presentation

Presentation and clinical history

A 28-year-old, third gravida with a history of two previous abortions was receiving routine antenatal care. Due to her obstetric history, a cervical cerclage was placed during the first trimester. She underwent regular radiological scans throughout her pregnancy. At 33 weeks of gestation, a radiological scan identified reduced amniotic fluid but showed no disparity in fetal growth. The patient was afebrile, with a pulse rate of 78 beats per minute and a blood pressure of 110/76 mmHg. Fetal heart sounds (FHS) were noted to be 142 beats per minute. Obstetric Doppler findings were within normal limits. The patient continued to be monitored at the OB/GYN clinic for amniotic fluid levels.

Investigations and diagnosis

**Figure 1:**
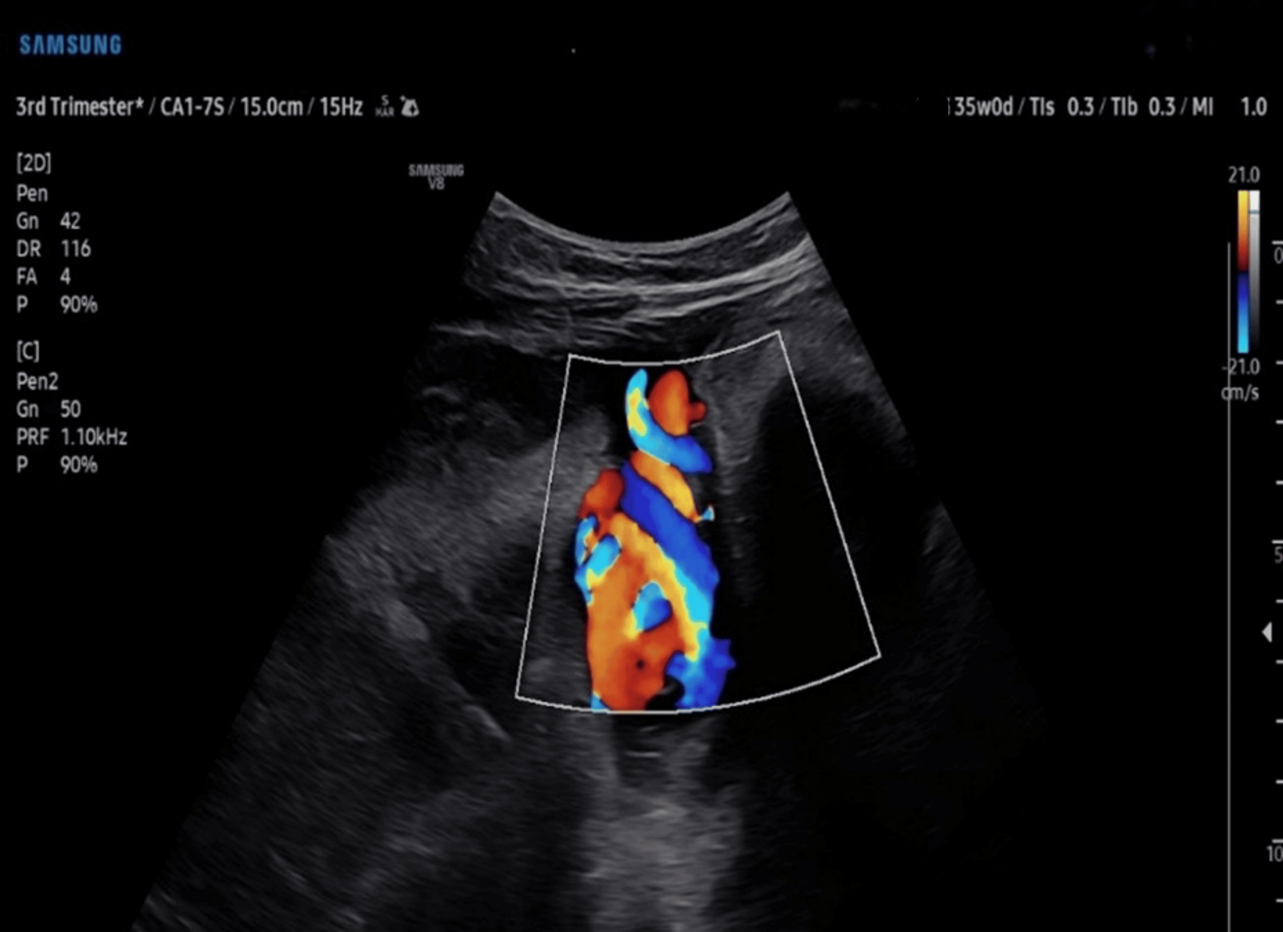
A true knot in the umbilical cord

**Figure 2:**
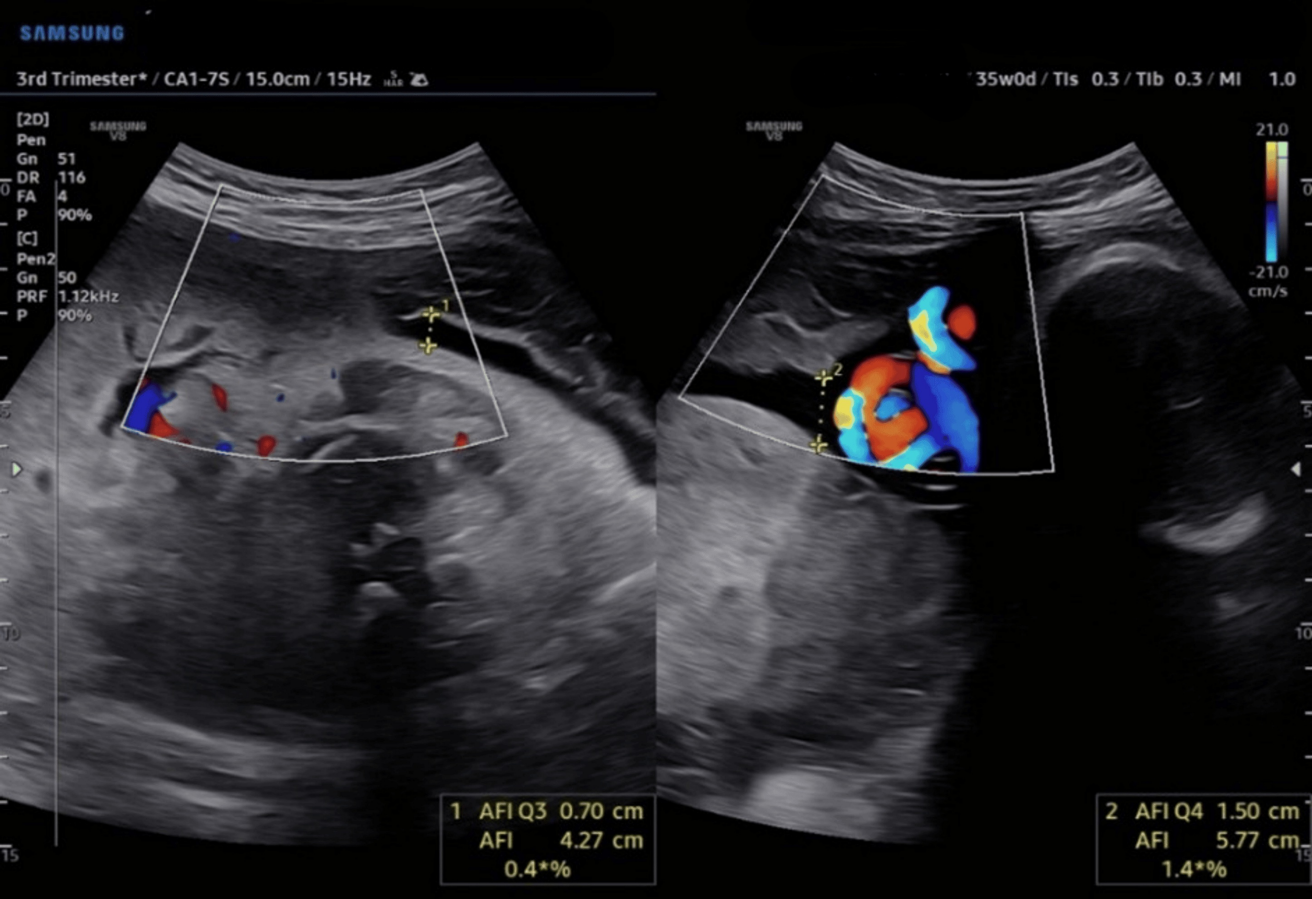
A true knot in the umbilical cord adjacent to the fetal head and AFI of 5.77 cm AFI: amniotic fluid index.

At 35 weeks of gestation, the patient was referred to radiology to reassess the amniotic fluid index (AFI), following a clinical suspicion of reduced fluid levels. The radiological scan revealed a borderline AFI of 5.7. Notably, the scan also identified a loop of the umbilical cord around the fetal neck and a true knot in the umbilical cord (as shown in Figures 1, 2). The obstetrician was immediately informed, and the patient who reported reduced fetal movements was advised to monitor fetal movements closely.

Treatment plan and surgical intervention

**Figure 3:**
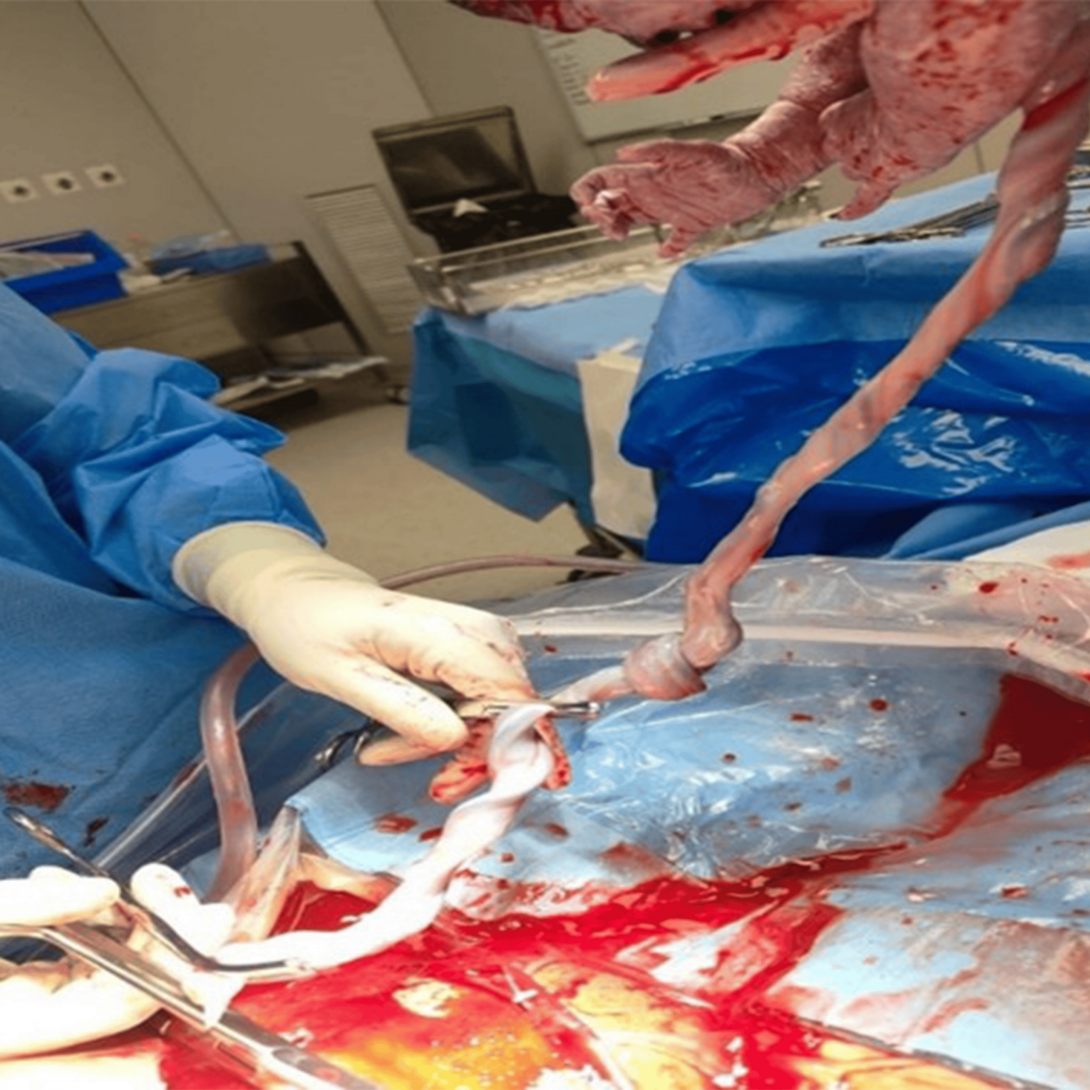
Intraoperative image showing surgical removal of the umbilical cord with a true knot and a long umbilical cord

Two days later, the patient presented with an absence of fetal movements for the past six hours. Cardiotocography (CTG) revealed suspicious findings, raising concerns about fetal well-being. An emergency lower segment cesarean section (LSCS) was performed with concurrent removal of the cervical stitch. Intraoperatively, a tight true knot in the umbilical cord was confirmed, consistent with the antenatal ultrasound diagnosis (as shown in Figure 3). A male baby weighing 2.29 kg was delivered in stable condition with Apgar scores of 9 at 1 minute and 9 at 5 minutes. The neonate was roomed in with the mother, and breastfeeding was initiated within the first hour.

Postoperative course

The postoperative period was uneventful. Recovery was normal, with the patient passing flatus and urinating normally on the first postoperative day. Bowel movements resumed after 34 hours. Bleeding per vagina (lochia) remained within normal limits. She was discharged 48 hours after delivery.

Follow-up

Follow-up after one week revealed that the skin incision had healed externally and the mother was breastfeeding normally. However, complete healing of the LSCS scar was achieved after several weeks.

## Discussion

Incidence and clinical relevance

TKUCs occur in approximately 0.3%-2.1% of pregnancies [1,3,5], with clinical outcomes ranging from benign to life-threatening. While a diagnosis of many cases is incidental and results in favorable outcomes [9], others are associated with serious complications such as intrauterine growth restriction (IUGR), fetal hypoxia, neurological impairment, and stillbirth [5]. Several studies have linked TKUCs to adverse outcomes like fetal demise [1,3,8]. These divergent findings underscore the influence of critical variables, including the degree of knot tightness [6], co-existing cords [10,11] or placental abnormalities [8], and other maternal-fetal conditions.

Several risk factors are associated with the formation of TKUC, including advanced gestational age, polyhydramnios, male fetus, long umbilical cord, multiparity, and a history of pregnancy loss [3,4,9,10]. Among which fetal male sex has been most consistently identified in the literature as a significant predisposing factor [1-8]. Our case highlights the significance of such contextual factors. The patient exhibited multiple risk factors for TKUCs, including multiparity, advanced gestation, and a male fetus; however, a borderline AFI of 5.7 was also noted, although it is not commonly among the typical risk factors like polyhydramnios, which has been associated with increased fetal mobility and knot formation. Subsequently, fetal movement cessation and CTG findings warranted an emergency cesarean delivery. 

Pathophysiology 

Increased fetal movement during early pregnancy is believed to contribute to the formation of TKUCs by promoting the looping of the cord [6]. However, adverse outcomes are more likely to manifest in the third trimester, when the natural decline in amniotic fluid volume limits cord mobility, increasing the risk of knot tightening [3]. One plausible, though unproven, hypothesis suggests that a TKUC may itself contribute to reduced amniotic fluid levels. A tightly formed knot can impair umbilical blood flow [3,5], potentially leading to decreased fetal renal perfusion and diminished urine output, an essential component of amniotic fluid volume regulation.

The umbilical cord serves as a vital conduit for oxygen, nutrients, and fetal circulation. The presence of a knot can impede venous return and compromise the delivery of oxygenated blood to the fetus, as reported by Waldron et al [8]. Partial obstruction of blood flow may lead to complications such as IUGR, while complete or acute compression can result in sudden fetal distress or even fetal demise [5]. 

Diagnostic limitations and imaging advances

Diagnosis of TKUC remains a significant clinical challenge; however, advances in imaging modalities such as 3D and 4D sonography, as well as color and power Doppler imaging, have improved detection rates [3,5]. A study conducted by Carter et al, has explored the potential of electronic fetal monitoring (EFM) parameters in detecting TKUC; however, the findings demonstrated limited correlation [7], rendering the diagnostic utility of EFM inconclusive.

Although 3D sonography, Color Doppler, and power Doppler have been suggested as potentially useful [3,6]; they are not definitive. These imaging techniques can sometimes yield false-positive results [1,6], and routine ultrasound often fails to detect many knots, increasing the risk of fetal complications. Given the limitations of current diagnostic tools and the potential for unnecessary interventions due to misinterpretation, there is a clear need for more accurate and validated imaging techniques to improve antenatal detection of TKUC.

Clinical management and follow-up considerations

Many authors have highlighted the absence of standardized guidelines for managing pregnancies complicated by TKUC [2,5,6,10], leading to variability in clinical practices. However, antenatal diagnosis of TKUC has been associated with improved fetal outcomes [2]. In our case, a multidisciplinary approach that included close fetal surveillance and early recognition of the TKUC facilitated timely intervention and resulted in a favorable neonatal outcome.

Risk stratification for pregnancies complicated by TKUCs should incorporate factors such as decreased amniotic fluid, multiple gestation, excessive cord length, male sex, multiparity, among others. When these risk factors are identified, a tailored monitoring plan may be necessary. Furthermore, educating patients on daily fetal movement counting may facilitate timely reporting of changes, as was observed in our case.

Postnatal evaluation of both the neonate and the mother is essential, particularly following TKUC-associated complications. Follow-up should include monitoring for any signs of neonatal hypoxia and other related complications. Additionally, counselling for the mother is important to address the potential implications for future pregnancies and provide appropriate guidance.

Although the information provided is based on incidental findings, our experience underscores the unpredictability of TKUCs, and the challenges associated with their antenatal diagnosis. While often benign, their presence carries uncertain perinatal risks. Detection alone should not alter the planned obstetric approach but warrants careful monitoring and preparedness. Although there are no standardized protocols for managing TKUCs, individualized care involving detailed patient counseling, daily fetal movement monitoring, and fetal surveillance is essential. Delivery is typically considered if signs of fetal compromise arise or if the pregnancy reaches term, rather than based on the presence of a true knot alone [6].

Clinical implications

This case reinforces the clinical complexity and potential severity of TKUC. While not all knots result in adverse outcomes, tight TKUCs, especially in the presence of coexisting risk factors, pose a significant threat to fetal well-being. Early identification through advanced imaging, such as 3D power sonography, is essential. Further research, particularly prospective studies and meta-analyses, is essential to identify effective diagnostic strategies and to establish evidence-based screening and management protocols for TKUC.

Maternal follow-up should focus on recovery from delivery and assessment of any obstetric complications associated with TKUC, such as placental insufficiency or abnormal uterine perfusion. Postpartum counselling regarding future pregnancy risks and the potential recurrence of TKUC can provide valuable guidance for affected mothers.

## Conclusions

This case highlights the critical role of advanced imaging and vigilant fetal monitoring in the antenatal detection of a TKUC, which directly contributed to a favorable perinatal outcome. Clinicians should maintain a high index of suspicion in high-risk pregnancies and utilize advanced Doppler imaging for early recognition. This case reinforces the importance of antenatal education, particularly regarding fetal movement monitoring. Encouraging patients to report reduced fetal activity can lead to early intervention, potentially improving neonatal outcomes. Future research should focus on establishing standardized diagnostic criteria and assessing the efficacy of advanced imaging techniques in TKUC detection and management
